# Coaching While Waiting for Autism Spectrum Disorder Assessment: Protocol of a Pilot Feasibility Study for a Randomized Controlled Trial on Occupational Performance Coaching and Service Navigation Support

**DOI:** 10.2196/20011

**Published:** 2021-01-07

**Authors:** Charmaine Bernie, Katrina Williams, Fiona Graham, Tamara May

**Affiliations:** 1 Department of Paediatrics The University of Melbourne Melbourne Australia; 2 Department of Allied Health The Royal Children's Hospital Melbourne Australia; 3 Murdoch Children's Research Institute Melbourne Australia; 4 Department of Paediatrics Monash University Clayton Victoria Australia; 5 Developmental Paediatrics Monash Children’s Hospital Melbourne Australia; 6 The Murdoch Children’s Research Institute Melbourne Australia; 7 Rehabilitation Teaching and Research Unit Department of Medicine University of Otago Wellington New Zealand

**Keywords:** coaching, Occupational Performance Coaching, feasibility, parents, caregivers, ASD, autism, waiting list, referral, service navigation

## Abstract

**Background:**

In Australia, the average time between a first concern of autism spectrum disorder (ASD) and diagnosis is over 2 years. After referral for assessment, families often wait 6-12 months before their appointment. This can be a time of uncertainty and stress for families. For some families, other forms of assistance are not accessible and thus timely intervention opportunities are missed. There is little evidence about how to provide the best support for children or caregivers while on assessment waiting lists.

**Objective:**

The aim of this study is to determine whether use of a coaching intervention called Occupational Performance Coaching (OPC) combined with service navigation support is feasible for families waiting for ASD assessment, as a crucial first step in planning a randomized controlled trial.

**Methods:**

A pilot and feasibility study will be conducted using recommended constructs and associated measures, which will be reported using CONSORT (Consolidated Standards or Reporting Trials) guidance. Participants will be child and caregiver dyads or triads, recruited within 4 months of their child (aged 1-7 years) being referred to one of two services for an ASD assessment in Victoria, Australia. A blinded randomization procedure will be used to allocate participants to one of three trial arms: (1) coaching and support intervention delivered face to face, (2) coaching and support intervention via videoconference, and (3) usual care. Descriptive statistics will be used to describe the sample characteristics of parents and children, inclusive of service access at baseline and follow up. Recruitment rates will be reported, and retention rates will be evaluated against a predicted rate of 70%-80% in each intervention arm. Goal attainment, using the Canadian Occupational Performance Measure, will indicate preliminary evidence for efficacy within the intervention arms, with an increase of 2 or more points on a 10-point performance and satisfaction scale considered clinically significant.

**Results:**

The study was approved by The Royal Children’s Hospital Research Ethics and Governance Department in September 2018. As of October 2020, 16 families have been recruited to the study. Data analysis is ongoing and results are expected to be published in 2021.

**Conclusions:**

Study findings will support planning for a future randomized controlled trial to assess the efficacy of OPC and service navigation support for caregivers of children awaiting ASD assessment.

**Trial Registration:**

Australian New Zealand Clinical Trials Registry ACTRN12620000164998; www.anzctr.org.au/Trial/Registration/TrialReview.aspx?id=378793&isReview=true

**International Registered Report Identifier (IRRID):**

DERR1-10.2196/20011

## Introduction

Autism spectrum disorder (ASD) is a wide-ranging developmental disorder defined by atypical social communication and behaviors [1]. In Australia, ASD can affect between 1% and 4% of children [2], and is typically diagnosed by pediatricians, psychologists, or psychiatrists, with or without the support of allied health clinicians such as speech pathologists and occupational therapists [3].

Despite the high prevalence and increased community awareness of ASD, there remains a paucity of assessment services to meet demand across Australia, including in Victoria [4,5]. Such challenges and service inequities have a significant impact on the early pathway for children and families [6]. Although a prompt diagnosis is made for some children after seamless recognition and assessment by service providers, many families wait years between identification of learning, social communication, and behavior differences and diagnostic assessment [7]. It is common for families to be referred to multiple service providers before an understanding of service needs is obtained, or to wait extended periods on waiting lists for assessment before accessing vital therapy services [8].

Australia, similar to other parts of the world, currently faces the challenge of balancing the need for comprehensive assessment against high demands for diagnostic services [9,10]. Alongside establishment of the Australia-wide, age-limited Helping Children with ASD Package (FACSIA funding) in 2008 [11], demand for early diagnosis has increased and placed notable pressure on assessment services [12]. In Australia, this funding model was recently replaced with the individualized, client-controlled National Disability Insurance Scheme, which oversees both early intervention and disability support services in the country. In the context of this changed funding model, publicly funded ASD assessment services remain under-resourced and overburdened [13]. This is likely due to recommended comprehensive diagnostic practices, as well as an ongoing emphasis on diagnosis as the entry point for further service access in some of Australia’s disability and education systems [13,14]. Moreover, state-wide initiatives in Victoria have recently been funded to improve the identification of ASD risk in young children via early screening initiatives, but without a matched expansion of publicly funded diagnostic services [15]. This is likely to increase the existing burden on assessment services in the coming years, further extending wait times and service access delays for children and families. The issues surrounding timely service delivery are not limited to either ASD assessment or to Australian settings, but rather represent an international health service issue in pediatrics; thus, calls for action to address these delays are continuing [16,17].

For families of children with an identified risk of ASD, emerging concerns are exacerbated by limited service availability and a lack of clarity regarding suitable interventions and support [13,18]. Outside of research focusing on siblings of children with ASD, who are known to have an elevated risk for the disorder, there is little research to date that addresses how a child’s and family’s needs are met, or not met, at this crucial stage when concerns first arise [19]. Despite this limitation, there is a general consensus among clinicians and researchers about the importance of timely access to needs-based services that have the potential to improve a child’s long-term outcomes [20] and the outcomes for their family.

In particular, few studies have explored ways to support children and families while waiting for ASD assessment services [16]. Moreover, no study to date has provided a rigorous, methodological approach to reviewing what interventions, if any, best address caregiver-identified needs for their child and family at this stage.

Coaching interventions have been investigated for primary carers seeking support because their young child is experiencing developmental difficulties [21-23]. A recent systematic review highlighted their general acceptability, although only 5 randomized controlled trials (RCTs) have been reported [24]. Along with methodological flaws, the wide-ranging definitions for coaching and its subtypes were described. Coaching diversity and inconsistent outcome measurement were reported to have impeded efficacy conclusions. Occupation-orientated coaching, which has been delivered via face-to-face and videoconference modalities, is one coaching subtype [24,25] in which fidelity measures have been proposed and published as a way of addressing identified intervention inconsistencies [26,27].

Occupational Performance Coaching (OPC) is an occupation-informed intervention developed to address the functional support needs of children and families [23]. OPC is theoretically informed by occupation and family-centered practice, along with ecological models of child health and well-being. This approach involves supporting families to generate goals relating to themselves, their child’s functioning, or the functioning of their family as a whole. The therapist, through a series of interactive semistructured interview sessions, then provides opportunities for reflection, sharing of knowledge, and the development of attainable actions. Performance analysis, strategy generation, and resource identification take place collaboratively, and actions are reviewed in subsequent sessions until goals are reached. Effectiveness of this intervention holds promise and continues to be evaluated [28-30]. To date, OPC has been typically examined in face-to-face sessions around 60 minutes in length, with a range of dosages between 2 and 12 sessions [31]. However, OPC has not yet been tested specifically with families of children waiting for ASD assessment, nor have the modes of treatment delivery, including delivery via telehealth or videoconference modalities, been compared concurrently within the same study. These modalities require particular exploration given social distancing challenges brought about as a result of the COVID-19 pandemic.

In novel clinical trial applications, the importance of initial feasibility testing and reporting has been reiterated in recent years [32,33]. Therefore, the aim of this study is to assess whether a small number of coaching sessions is feasible for families and has potential to address family needs while waiting for ASD assessment. The intervention’s acceptability, practicality, expandability, and demand, as well as the types of needs in the local context that require interventions [34] will be examined. Such explorations are essential to inform a future randomized clinical trial protocol, with risk of bias minimization and appropriate methodological rigor.

The specific aims of this study are: (1) to assess the feasibility, including constructs of acceptability, practicality, and preliminary efficacy, of an RCT study design exploring goal-directed support for families of children waiting for ASD assessment; and (2) inform protocol planning for a future RCT to assess efficacy of short-phase coaching and family support via face-to-face and videoconference modalities.

It is envisaged that the combined results from this feasibility study and a future RCT will inform service planning as well as service standards to address the needs of families with a child waiting for ASD assessment. The findings may also support new methods to eradicate wasteful waiting periods and prevent clinical practices that hinder access to needs-based interventions.

## Methods

### Trial Design

**Figure 1:**
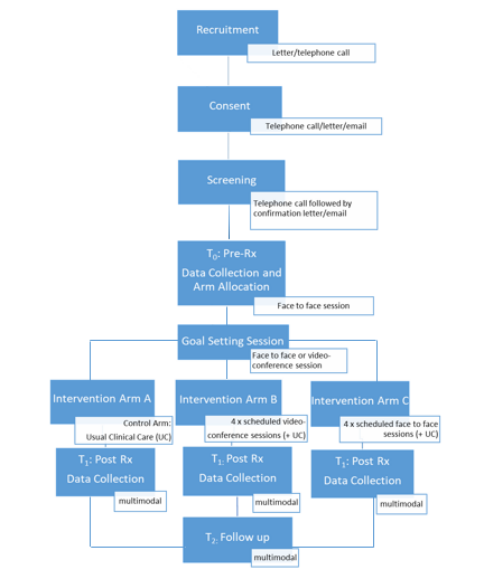
Study procedures flow chart.

Trial design elements are described in line with CONSORT (Consolidated Standards of Reporting Trials) guidance for pilot and feasibility studies [35]. This study is a pilot RCT and feasibility study, with a focus on feasibility outcomes, following participant allocation to one of three parallel study arms inclusive of a usual care group. The study flow is detailed in Figure 1.

Allocation ratios will be determined by the randomization process outlined below with an aim of obtaining an even number of participants allocated to each study arm.

### Participants

Children below 7 years of age referred to the Royal Children’s Hospital (RCH) in Melbourne for an autism assessment and their primary caregivers will be recruited for the study. Approximately 50 children per month are referred to the RCH by internal or external service providers for the specific purpose of ASD assessment. Referrals are typically received from all over the state of Victoria, although geography can be an exclusion criterion for some of the assessment services. Additionally, Melton Health provides services for children and their families in the western suburbs of Melbourne, Victoria, and also receives high volumes of referrals per month. In both services, children are typically triaged centrally by a single, clinically trained ASD service coordinator. Referrals are screened for their appropriateness for services based on available clinical information, including demographic information such as home address, identified referral concerns, previous service history, and referrer background.

The outcome of triage may be acceptance to a single professional group or multidisciplinary team for assessment to other developmental, medical, or behavioral services within the hospital, or redirection to an appropriate external service. Decision making occurs in line with specific service eligibility criteria, family resources, and service availability, and the nature of the presenting concerns.

The inclusion criteria are: (1) child up to the age of 6 years, 11 months with a recent (within the last 4 months) and active referral received querying ASD, who lives at home with primary caregivers; and (2) child and primary caregiver dyads or triads, which may include the child and up to two primary caregivers who live at home with the child. The exclusion criteria are: (1) child already diagnosed with ASD at the hospital or at an external service; (2) any participating primary caregiver who is unable to provide informed consent at the time of recruitment or at baseline (T_0_); (3) any participating primary caregiver who is currently accessing regular (weekly or biweekly) coaching or counseling support with a health professional relating to the care of their child or their individual mental health needs; and (4) child is aged 7 years or older at the time of referral.

### Recruitment and Consent

Following service allocation at the conclusion of referral triage at a tertiary hospital in Melbourne who accepts referrals for autism assessment, a study information leaflet will be sent by post to eligible families via the clinical service coordinator. Families who respond to the letter via telephone, email, or letter will proceed to the screening and consent stage. Families for whom no response is received will be telephoned by the triage clinician to enquire about the receipt of the leaflet and ascertain interest in the study. No more than two attempts will be made to contact nonresponding families.

For those who communicate interest in the study via telephone, email, or mail, screening will occur via a telephone-based interview conducted by the principal investigator. Eligibility will be determined by both the caregiver report, and information contained in the referral letter and the child’s medical record. If inclusion criteria are met and no exclusions identified, participants will be sent the consent form and parent information statement to complete. Once a signed form is received, a face-to-face appointment to complete baseline study measures (T_0_) will be made.

### Interventions

In the first study arm (A), families will receive usual clinical care. Usual clinical care consists of telephone or email access to an ASD triage and service coordinator during the clinician’s working hours 2 days per week. This clinician is based at a tertiary children’s hospital for service direction and advice as needed, and has relevant expertise in ASD and developmental service delivery at a senior clinician level. Duties include answering service–related queries, offering advice regarding symptom presentation and management, and providing telephone-based counseling as required. Usual care also includes any local service provision that the child or parent may be accessing to address previously identified developmental, behavioral, or health-related issues. These may include, but are not restricted to, access to a local pediatrician, speech pathologist, occupational therapist, or psychologist. Findings from the feasibility study will help to further inform community-based usual care provisions while children await autism diagnostic services.

In addition to usual care, OPC [31] will be carried out with participants in the other two study arms (B and C), differing only by mode of intervention delivery (videoconference vs face-to-face coaching). For the purpose of this study, videoconference is defined as an encounter between the intervention provider and study participants via a live video and audio link.

The intervention arms will be delivered by a clinician who is an experienced pediatric occupational therapist. Occupational therapists are increasingly part of child neurodevelopment assessment and intervention teams, supporting families to identify goals that will assist their child’s functions and participation and improve family quality of life. They are well trained to support families to choose interventions, and to engage children and families in interventions that can help them achieve meaningful or functional goals [36].

In OPC, participants will initially be supported to identify goals using the Canadian Occupational Performance Measure (COPM) and additional questions that frame the vision of the goal. Goals identified in OPC are expressed as personally valued activities or routines in the contexts of daily life (ie, home, school, or community settings). For example, a parent’s goal may be for the child and their family to collect groceries at the local grocery store once per week. Once goals are identified, the intervention provider will then use guiding questions and reflection techniques, including prompts or probes that help the caregiver to explore ideas and possible solutions. OPC will be subsequently delivered as per the approach’s training manual [31].

Participants in arms B and C will be invited to attend 4 sessions of OPC. These will be 45-60–minute sessions, held between 1 and 5 weeks apart, depending on the participant’s preference. This is to ensure there is adequate time between sessions to support strategy implementation outside of the intervention session itself, while meeting families’ variable attendance needs. Participants allocated to the videoconference arm will be able to identify their preferred app for connecting with the intervention provider, which may include (but not be limited to) Zoom, WhatsApp, or Skype apps.

The delivery of OPC will be monitored using an established fidelity tool [37]. Intervention will be delivered by a clinician who has attended 24 hours of face-to-face training and further support hours relating to intervention fidelity, conducted by the original author of OPC. For every 3 participants that enroll in the study, all of the audio tapes from 1 participant will be submitted to the author of OPC for fidelity measurement. The first set of audio recordings will be used to develop familiarity with the fidelity measure and will not be included in the final fidelity analysis. Approximately 30% of the overall recordings in the study will be double-coded using the fidelity checklist by the author or associates trained in use of the fidelity measure, in addition to the trained investigator delivering the intervention. This will occur until 80% or more fidelity constructs have occurred for 4 participants in a row.

### Outcomes

#### Primary Feasibility Outcome Measures

Feasibility constructs adapted from Bowen et al [34] will be used to guide measures that will be reported following this feasibility trial. These will be measured as described in Table 1. In particular, recruitment and retention rates will be explored and reported, as well as tolerance of randomization and feasibility of collection of the preliminary efficacy measures described in Table 1. Two standardized measures, the COPM [38] and the Measure of Processes of Care-20 [39], will also be used pre- and postintervention to assist with measurement of these feasibility constructs.

**Table 1:** Primary feasibility outcome measures.

Feasibility construct	Recruitment rate	Retention rate	Goal attainment (COPM^a^) [38]	Measure of Processes of Care-20 [39]	Postintervention questionnaire	Time, resource, cost analyses (posthoc)
Acceptability	x	x		x	x	
Demand	x	x				
Implementation	x	x	x			x
Practicality			x	x		x
Adaptation					x	x
Integration Expansion	x	x		x		x
Limited-efficacy testing			x		x	

^a^COPM: Canadian Occupational Performance Measure.

All families will have goals set using the COPM. Although listed in Table 1 as an outcome, the process of goal setting and engagement in the COPM is additionally considered to be an intervention by some researchers and clinicians [40].

#### Secondary and Preliminary Efficacy Outcome Measures

Characteristics of the participants, inclusive of the child and the caregiver(s), will be collected at baseline (T_0_). Secondary measures will also be collected at T_0_ and at follow up (T_1_) to assess participants’ current priorities for the intervention, services accessed, and perspectives on service provision. Some measures will serve the dual purpose of measuring preliminary efficacy of the intervention (Table 2).

**Table 2:** Secondary preliminary efficacy measures.

Measure	Areas assessed and assessment duration	Psychometric properties and time points for measurement
Vinelands Adaptive Behavior Scales 3 (VABS 3) [41]	Adaptive behavior and general functioning: child; 20 to 60 minutes	Validity and reliability established in children with developmental disabilities or ASD^a^ [42]. To be administered at T_0_^b^ and T_1_^c^
Social Responsiveness Scale (SRS) [43]	Child’s social communication skills and ASD symptoms; 20 minutes (for children older than 2.5 years)	Reliability and validity established internationally [44,45]; good construct validity and internal consistency found. To be administered at T_0_ and T_1_
Parenting Stress Index (Short) [46]	Caregiver stress as it relates to the child with presenting difficulties; 10 minutes	Well-established psychometric properties in various populations, including high-risk mothers and infants [47], and parents of toddlers in low-income areas [48]. To be administered at T_0_ and T_1_
Beach Centre Family Quality of Life Scale [49]	Family quality of life; 5 minutes	Reliability and validity established in families of children with disabilities [50]. To be administered at T_0_ and T_1_
Parent and Child History Questionnaire (self-designed)	Parent and child history; 10 minutes	Administered at baseline (T_0_) with questions regarding current services accessed. To be administered at T_0_ and T_1_

^a^ASD: autism spectrum disorder.

^b^T_0_: baseline.

^c^T_1_: follow up.

### Data Collection

All written forms completed by study participants, standardized and nonstandardized, will be explained in full to each participant prior to completion at T_0_ and T_1_. They will be checked for accuracy and completeness, with any issues that emerge clarified, and managed by the principal investigator. Data collection during intervention sessions will be via direct audio recording using a dictaphone or equivalent recording device, or video recording.

In addition to source data collected at the first and last study visit, data will be collected directly from assessment teams regarding the diagnostic assessment, following signed permission from the study participant. Diagnostic assessment information will include clinical report and chart reviews, and are included in Figure 1 as T_2_.

Electronic study records will be stored on a password-protected, reidentifiable database in REDcap [51], backed up on a secure server. Paper records will be securely stored as per the study’s ethics approval process.

### Sample Size

A pragmatic sample size of 18-24 families is sought to allow for 6-8 participants randomized to each intervention arm. These numbers are in line with similar pilot and feasibility studies in relation to coaching, including those delivered via videoconference or telehealth [25,52]. Based on clinical and research expertise across the team, this number was considered to be sufficient to inform protocol planning related to a future RCT, and to satisfactorily meet the requirements for obtaining all research aims. With a sample size of 24, we will be able to predict a participation rate of 20% to within a 95% CI of 16% [53].

### Randomization and Blinding

#### Sequence Generation

Following initial recruitment actions carried out by the clinical service coordinator, the study’s principal investigator will complete the consenting process described above for arm allocation. Participant dyads or triads will then be randomly allocated to an intervention arm A, B, or C, where arm A is usual care, arm B is face-to-face coaching, and arm C is coaching via videoconference (Figure 1). Randomization will occur using a random allocation sequence generated in Microsoft Excel for each reidentifiable participant number, allocated per participant dyad or triad at study enrollment. The sequence will be executed by a blinded research team member who is not directly involved in screening or intervention provision.

#### Allocation Concealment Mechanism

Randomization and study arm allocation will occur prior to baseline measure commencement, and communicated via email or telephone to the participant and principal investigator (who is also the intervention provider) at the conclusion of baseline measure completion. It is not possible to conceal allocation to either party thereafter given that both the intervention provider and participant groups will be aware of either the absence or mode of intervention delivery. Follow-up (T_1_) measures will be completed by the principal investigator.

### Statistical Methods

#### Data Analysis

For the purpose of primary feasibility-related analyses, quantitative data will be analyzed with qualitative analyses relating to individual sessions and therapeutic progress conducted separately. Data analysis will occur according to the data measurement plan described above in Table 1 and Table 2. Descriptive statistics will be used to describe the sample characteristics of parents and children. Recruitment rates will be reported, and retention rates will be evaluated against a predicted rate of 70%-80% in each intervention arm [54]. Goal attainment, measured by performance and satisfaction ratings using the COPM, will provide preliminary evidence for efficacy within the intervention arms. An increase of 2 or more points on a 10-point performance and satisfaction scale in the COPM is considered clinically significant [55].

#### Power

Data gathered from this feasibility study will be used to calculate recruitment and retention rates. Primary and secondary outcome measure data will be used to inform future power calculations, which are required to estimate appropriate recruitment numbers for a fully powered RCT aimed at gathering efficacy data.

### Ethics Approval and Consent to Participate

All study attributes will be carried out in line with the National Health and Medical Research Council Act 1992, and following approval from the RCH Human Research Ethics Committee. Ethical approval was granted by The RCH Research Ethics and Governance Department in September 2018 (HREC 38154A), spanning all elements detailed in this protocol to take place across the campus organizations of The University of Melbourne, The Murdoch Children’s Research Institute, and the RCH, Melbourne. Only families who provide explicit consent to participate with a signed consent form will be eligible for this study.

## Results

The trial has concluded recruiting families of children referred for an ASD assessment throughout 2019. It remains under ethical approval and will continue throughout 2020. As of October 2020, 16 families have been recruited to the study.

Results will be reported according to CONSORT guidance [56] following the conclusion of this study. Feasibility findings as described above will be reported as primary outcomes. Preliminary efficacy findings will be reported as primary and secondary outcomes, as described in Tables 1 and 2.

## Discussion

To our knowledge, this is the first study measuring the feasibility of OPC via two different parallel modalities for families awaiting ASD assessment. Given the lag time between identification of concerns and assessment that many families experience, identification of an intervention that addresses primary family concerns in this interim period is warranted

Uncertainty exists in relation to optimal feasibility study methodology when testing new interventions or existing treatments for alternative populations. In recent years, efforts have been made to make recommendations regarding pilot and feasibility studies; however, there remains no consistent approach as to how such studies should be conducted and measured [34,54]. As such, we have incorporated a diverse range of feasibility constructs and outcomes to ensure comprehensive evaluation that can inform future trial planning.

Additional strengths of this study include broad participant inclusion, consideration of alternative modes of service delivery in comparison with usual care, and incorporation of fidelity measures. Exclusion criteria have been kept to a minimum to allow the research team to have a broad sense of the nature of families who are interested in, and able to complete, the intervention. In particular, the availability of an interpreter service is aimed at encouraging participation of families for whom English is their second language. Moreover, the study will measure the feasibility of two different modes of service delivery in comparison with usual care, and explore the tolerance of randomization to these study arms. Finally, the fidelity of the intervention will be measured using a published tool as described previously [37].

In spite of these strengths, there are several limitations to consider during interpretation of the feasibility results. In line with suggested general pilot and feasibility methodologies, the sample size will be smaller than that used in full-scale clinical trials or efficacy studies. Preliminary efficacy findings will only be used as an aid to plan for a future RCT. Nevertheless, the planned sample size is consistent with similar studies that have focused on coaching, telehealth, or videoconference interventions [25,52], and is appropriate to address the research aims.

Although a broad participant recruitment strategy has been formulated, occurring across two sites, data collection and face-to-face coaching sessions will be carried out at only one site (RCH). This may prove to be a limitation in either recruitment or retention. Additionally, given that these sites offer publicly funded services, the participant sample has the potential to be biased, likely excluding families for whom a prompt, private service for assessment was accessible. Such predictions are in line with previous research in publicly funded developmental assessment services in Australia [57], in which the researchers found that families accessing such services were more likely to have a lower sociodemographic status or have English as a second language when compared to the general population. Given our plan to include families who may require interpreting services, translation and tool validation–related issues will mean that the secondary feasibility measures will need to be interpreted with caution. A future RCT will likely require coaching to be delivered at multiple sites to maximize the recruitment and generalizability of results.

Finally, all families who are participating in the study, regardless of study arm allocation, will continue to receive usual care. This is likely to be highly variable given that service access depends on family resources, geographical location, and other factors not yet known to the research team. Usual care will be described and considered in relation to relevant findings.

This study is the first step toward addressing an evidence gap by exploring potential interventions that can support families when on an ASD assessment waiting list. Findings of this study could inform the best care for families and children on waiting lists for other presentations, catering to an ongoing issue in health systems internationally.

## Appendix

Multimedia Appendix 1 CONSORT 2010 checklist.

## Abbreviations

ASD: autism spectrum disorder
CONSORT: Consolidated Standards of Reporting Trials
COPM: Canadian Occupational Performance Measure
OPC: Occupational Performance Coaching
RCH: The Royal Children’s Hospital, Melbourne, Australia
RCT: randomized controlled trial
